# Radiomics-based multiple machine learning approaches for investigating medial wall invasion of the cavernous sinus in pituitary adenomas

**DOI:** 10.3389/fonc.2025.1706895

**Published:** 2025-11-20

**Authors:** Yuyang Chen, Jiansheng Zhong, Pengwei Hou, Xiaoyu Wang, Jun Li, Ziqi Li, Tianshun Feng, Liangfeng Wei, Yuhui Chen, Shousen Wang

**Affiliations:** 1 Department of Neurosurgery, Fuzong Clinical Medical College of Fujian Medical University, Fuzhou, Fujian, China; 2 Fujian Provincial Clinical Medical Research Center for Minimally Invasive Diagnosis and Treatment of Neurovascular Diseases, Fuzhou, Fujian, China; 3 Department of Neurosurgery, Huashan Hospital, Fudan University, Shanghai, China; 4 Department of Neurosurgery, Shanghai Sixth People’s Hospital Fujian, Jinjiang, China; 5 Department of Neurosurgery, Fuzhou Changle District People’s Hospital, Fuzhou, Fujian, China

**Keywords:** pituitary adenoma, dural invasion, medial wall of cavernous sinus, machine learning, 3D slicer

## Abstract

**Objective:**

This study aims to develop a predictive model for cavernous sinus dural invasion in pituitary adenomas by retrospectively analyzing clinical and imaging data. It explores the associations between clinical and radiomics features and cavernous sinus dural invasion.

**Methods:**

Clinical data and coronal T2-weighted MRI images were collected from patients diagnosed with pituitary adenomas at our institution between December 2012 and December 2022. Tumor regions of interest (ROIs) were segmented using 3D Slicer, and radiomics features were extracted. Statistically significant radiomics features were identified using Lasso regression and univariate analysis. Clinical features were screened using univariate and multivariate logistic regression analyses. These selected features were incorporated into ten machine learning algorithms to construct three predictive models: a clinical feature model, a radiomics feature model, and a combined clinical and radiomics feature model. Model performance was evaluated to determine the best-performing model, which was further interpreted.

**Results:**

A total of 252 patients with histopathologically confirmed pituitary adenomas were included. The analysis identified Knosp grade, tumor left-right diameter, pedunculated satellite tumor, and clival invasion as significant clinical predictors, along with radiomics features including original.4, original.10, log-sigma-5-0-mm-3D.29, log-sigma-5-0-mm-3D.91, wavelet-LLH.37, wavelet-LHL.37, and wavelet-HLL.8. The combined clinical and radiomics model outperformed models based solely on clinical or radiomics features. Among the ten machine learning algorithms, the LightGBM model demonstrated the best predictive performance, achieving an area under the curve (AUC) of 0.86 and an accuracy (ACC) of 0.76.

**Conclusions:**

A machine learning model integrating clinical and radiomics features can effectively predict cavernous sinus dural invasion in pituitary adenomas preoperatively, providing a reliable basis for diagnosing tumor invasiveness and developing surgical plans. The LightGBM algorithm exhibited the highest predictive efficacy. Furthermore, the pedunculated satellite tumor feature emerged as a novel imaging marker for cavernous sinus dural invasion, offering new insights into the study of invasive pituitary adenomas.

## 1 Introduction

Pituitary adenomas are benign tumors originating from the anterior pituitary gland, accounting for approximately 15–20% of all intracranial tumors (1, 2). These tumors typically grow expansively, invading surrounding tissues and exerting mass effects that lead to neurological symptoms (3). The sellar region, surrounded by transitional dura mater, is particularly vulnerable to such invasion (4). As pituitary adenomas grow invasively, tumor tissue can compress and breach the adjacent dura mater, potentially extending into structures such as the suprasellar region and cavernous sinus (5). Early studies identified dural invasion as a key criterion for determining tumor invasiveness (6, 7), making the assessment of dural invasion a primary focus in the study of pituitary adenomas’ aggressiveness.

The cavernous sinus consists of five walls, with the medial wall, a single-layered dura separating it from the pituitary gland, being particularly significant in the context of invasion. Anatomical defects in some individuals may contribute to cavernous sinus invasion (8). Medial wall invasion of the cavernous sinus has long been considered a hallmark of aggressive pituitary adenomas. Radiological detection of medial wall involvement is an important preoperative method for evaluating cavernous sinus dural invasion. However, despite high-resolution T2-weighted imaging, the medial wall of the cavernous sinus remains difficult to visualize clearly (9). The 3D-SPACE sequence has emerged as one of the most effective MRI techniques for evaluating the dura mater due to its unique imaging properties (10). However, its long acquisition time and high cost limit its widespread clinical application. Therefore, accurate assessment of cavernous sinus dural invasion using standard MRI sequences remains a critical area of investigation. The “pituitary adenoma dural invasion channel theory” has been proposed, along with novel markers such as “pedunculated satellite tumors” and “interdural tumor,” to facilitate the preoperative assessment of cavernous sinus dural invasion in pituitary adenomas (11, 12). Their study indicated that intraoperative complete resection of the invaded medial wall of the cavernous sinus significantly affects the recurrence of pituitary adenomas. However, routine resection of the medial wall of the cavernous sinus during surgery may substantially increase the risk of intraoperative hemorrhage, thereby compromising surgical quality and postoperative recovery. Therefore, accurate preoperative prediction of medial wall invasion of the cavernous sinus can provide valuable guidance for neurosurgeons in determining whether to perform resection of the medial wall. Beyond cavernous sinus dural invasion, surgical complexity in pituitary surgery is also influenced by tumor consistency. Recent studies have introduced radiological surrogates of consistency—most notably the T2-weighted signal intensity ratio (T2SIR)—and shown that firmer tumors (lower T2SIR) are associated with reduced odds of gross-total resection and greater operative difficulty in non-functioning pituitary adenomas (13, 14). These advances contextualize the present work, which specifically targets preoperative identification of cavernous sinus dural invasion using conventional T2-weighted imaging and radiomics.

Building on this foundational research, our study focuses on MRI T2-weighted imaging to investigate cavernous sinus dural invasion in pituitary adenomas. Radiomics involves the analysis and processing of medical images, such as CT, MRI, and PET scans, to extract radiomic features used for screening, diagnosis, follow-up, and prognosis (15, 16). The integration of radiomics with machine learning has become a critical approach in clinical research. In this study, multiple machine learning algorithms were applied to analyze and extract radiomic features from coronal T2-weighted images of pituitary adenomas. A preoperative predictive model for cavernous sinus dural invasion in pituitary adenomas was developed to support the assessment of tumor invasiveness and inform surgical planning (17, 18).

## 2 Method

### 2.1 Study subjects

Clinical and imaging data were consecutively collected from patients who underwent transsphenoidal pituitary adenoma resection under microscopy at the Department of Neurosurgery in our hospital between December 2012 and December 2022. A total of 252 cases were included in the analysis after applying strict inclusion and exclusion criteria. Although consecutive sampling was used over a 10-year period, all cases were obtained from a single tertiary medical center, which may limit the generalizability of our findings. Selection bias could not be entirely excluded, as only patients with complete imaging and surgical data were included. However, consecutive case inclusion helps to reduce subjective selection and reflects real-world clinical practice. This study was approved by the Ethics Committee of the 900th Hospital of the People’s Liberation Army Joint Logistic Support Force in Fuzhou, Fujian, China (Approval Number: Ethics Review No. 2024-006). All procedures followed the principles outlined in the Declaration of Helsinki. Written informed consent for the reuse of general data was obtained from all participants during their hospital stay.

#### 2.1.1 Inclusion criteria

The inclusion criteria were as follows: 1) Complete imaging and clinical data; 2) Underwent transsphenoidal pituitary adenoma resection at our hospital; 3) Postoperative pathology and immunohistochemistry confirmed the diagnosis of pituitary adenoma.

#### 2.1.2 Exclusion criteria

The exclusion criteria were as follows: 1) Patients who received preoperative or postoperative cranial radiotherapy; 2) Patients with a history of sellar region surgery or medication for pituitary conditions; 3) Incomplete clinical or imaging data; 4) Coexisting brain trauma, meningitis, brain abscess, or cerebrovascular diseases; 5) Coexisting intracranial multiple tumors or malignancies. After rigorous screening, 252 cases were included in the analysis, comprising 132 males and 120 females.

### 2.2 Variable selection

In our study, demographic information and disease-specific characteristics were collected based on a review of the literature. Data were extracted from patients’ electronic medical records. Potential risk factors for cavernous sinus dural invasion of pituitary adenomas were identified based on published data, clinical expertise, and practical considerations for future clinical implementation.

**Figure 1:**
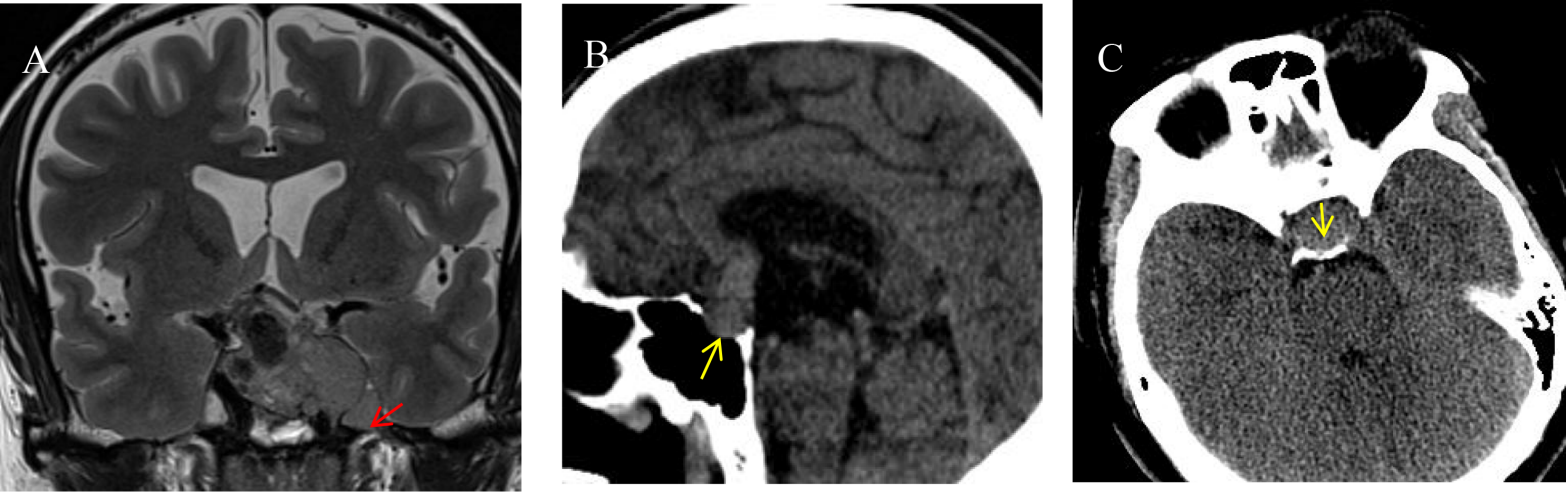
Imaging markers on different sequences. **(A)** Pituitary adenoma invading the left cavernous sinus is visible on an MRI T2-weighted image. A pedunculated satellite tumor (indicated by the red arrow) protrudes from the main tumor. The satellite tumor is clearly demarcated from the primary tumor, encapsulated, and exhibits characteristics of benign growth. **(B)** A sagittal CT image shows the posterior wall of the sphenoid sinus invaded by the pituitary tumor (indicated by the yellow arrow). The tumor breaches the posterior wall and extends into the sphenoid sinus cavity. **(C)** An axial CT image reveals the pituitary tumor invading the clivus (indicated by the yellow arrow). The clivus shows irregular, indistinct edges, with thinning of the bony structure.

Clinical data (19, 20) included gender, age, height, weight, BMI, and obesity classification (21) (categorized as 1 for lean, 2 for underweight, 3 for overweight, and 4 for obese). Imaging data (22) included tumor height, tumor anteroposterior diameter, tumor left-right diameter, tumor volume, Knosp classification (categorized as 1 for grades 0–1, 2 for grades 2–3a, and 3 for grades 3b–4), pedunculated satellite tumor (**Figure 1A**), tumor cystic change, tumor apoplexy, sphenoid sinus invasion (**Figure 1B**), and clival invasion (**Figure 1C**).

**Figure 2:**
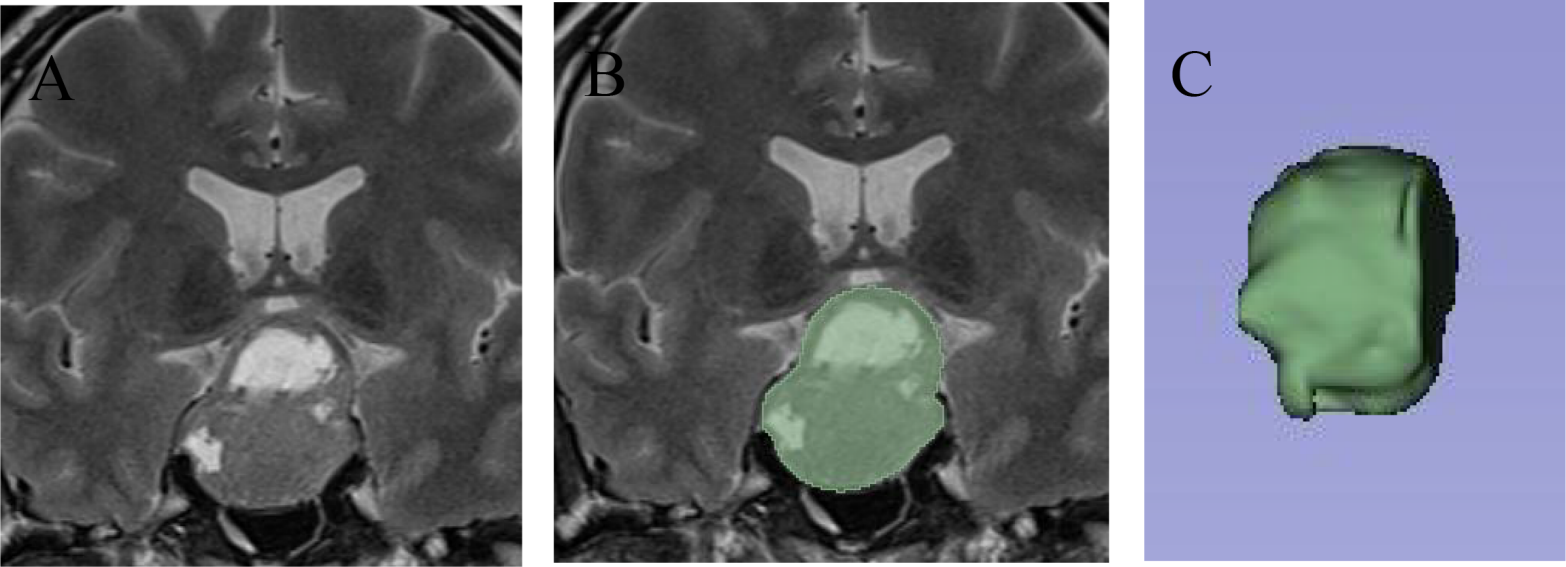
Delineation of the pituitary adenoma ROI using 3D slicer. **(A)** On T2-weighted imaging, a large pituitary adenoma is seen invading the suprasellar region and cavernous sinus, with cystic changes within the tumor. **(B)** The 3D Slicer tool was used to outline the full-layer ROI of the pituitary adenoma, showing the complete boundaries of the tumor. **(C)** After ROI delineation, a three-dimensional reconstruction was performed to visualize the tumor’s spatial structure.

**Figure 3:**
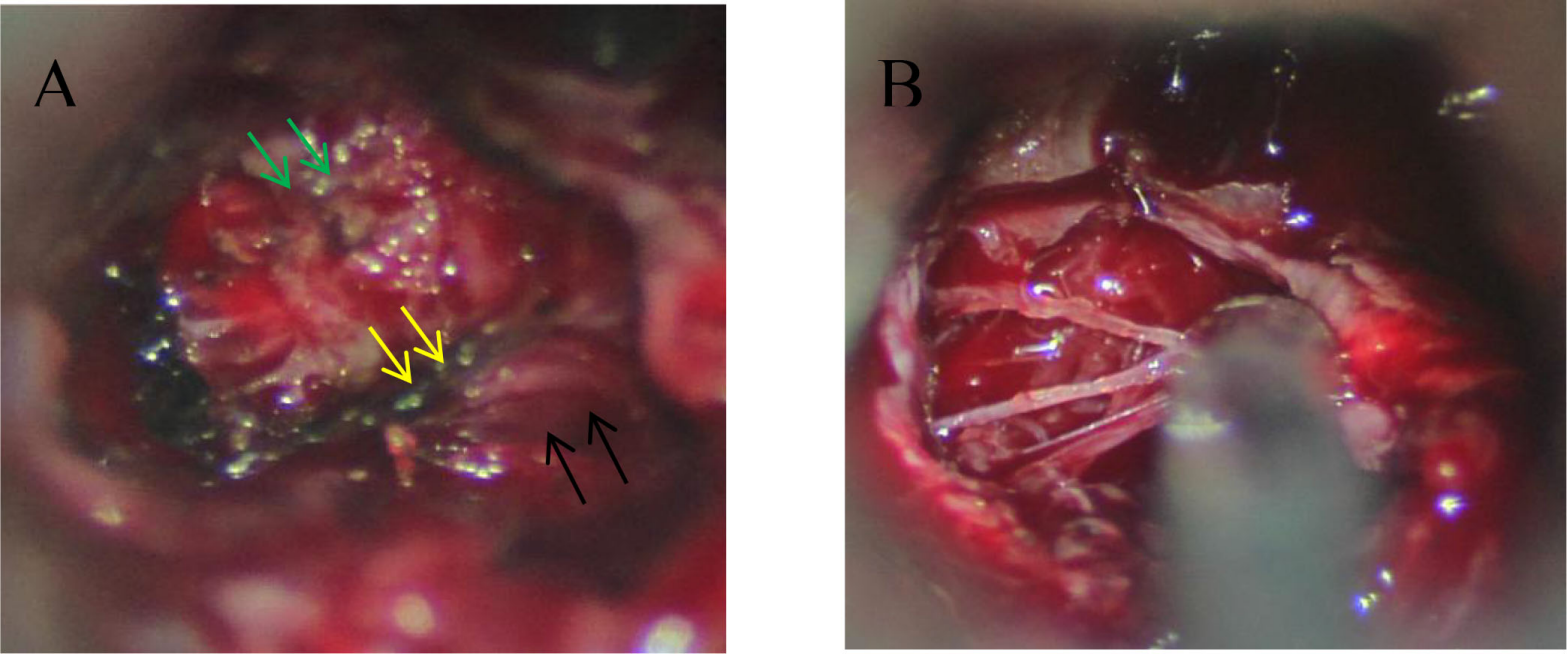
Intraoperative findings of dural invasion. **(A)** During microscopic transsphenoidal resection of a pituitary adenoma, the pituitary fossa (black arrow), cavernous sinus (green arrow), and medial wall of the cavernous sinus (yellow arrow) are clearly visible, showing well-defined structures. **(B)** Intraoperative observation reveals that the medial wall of the cavernous sinus has been disrupted by the tumor, leaving only a thin collagen fiber mesh. The integrity of the dura mater is significantly compromised.

Radiomics features were extracted using 3D Slicer by two radiologists and one neurosurgical attending physician who independently outlined the regions of interest (ROI) on coronal T2-weighted images(**Figure 2**). To assess inter-observer variability, 30 randomly selected cases were segmented by all three observers, and the Dice similarity coefficient (DSC) was calculated to evaluate agreement. Consensus was reached for the final segmentation through discussion. A variety of radiomic features—including first-order statistics, texture features (GLCM, GLDM, GLRLM, GLSZM, NGTDM), and shape descriptors—were extracted. Feature selection was based on variance, correlation, and SHAP importance scores. Wavelet-based features were retained due to their ability to capture directional and high-frequency texture patterns relevant to tumor invasion. The dataset was divided into training (70%) and testing (30%) sets using stratified sampling to ensure balanced representation of invasive and non-invasive cases. The gold standard for cavernous sinus dural invasion in this study was determined by intraoperative identification of defects in the medial wall of the cavernous sinus (**Figure 3**).

### 2.3 Data preprocessing and model construction

The dataset of 252 patients was randomly divided into a training set (70%) and a test set (30%). Prior to model construction, clinical data were analyzed. Chi-square tests (for categorical variables) and nonparametric tests (for continuous variables) were applied to assess the significance of each variable (*P* < 0.05), identifying initial clinical predictors associated with cavernous sinus dural invasion. These selected clinical variables were further evaluated using univariate logistic regression analysis to confirm their statistical correlation with cavernous sinus dural invasion. For radiomics features, data standardization and dimensionality reduction were first performed. The Lasso regression algorithm was then applied to identify representative radiomics features. Subsequently, the selected features underwent univariate logistic regression analysis to filter statistically significant radiomics predictors. After feature selection, ten representative supervised machine learning algorithms were employed to construct predictive models: Decision Tree (DT), Random Forest (RF), Logistic Regression (LR), K-Nearest Neighbor (KNN), Support Vector Machine (SVM), Naive Bayes (NB), Light Gradient Boosting Machine (LightGBM), Adaptive Boosting (AdaBoost), Extreme Gradient Boosting (XGBoost), and K-Means Clustering (K-Means). To evaluate model stability and predictive power, 10-fold cross-validation was conducted. Specifically, the dataset was randomly divided into ten subsets, with nine subsets used as the training set and one as the validation set in each iteration, repeated ten times. The predictive outcomes from the cross-validation process were compared to assess model reliability. Upon model construction, the test set was used to evaluate the performance of each model. Metrics including accuracy (ACC), true positive rate (TPR), false positive rate (FPR), positive predictive value (PPV), F1 score (FSC), sensitivity (SEN), specificity (SPE), and negative predictive value (NPV) were calculated and compared. Receiver operating characteristic (ROC) curves were plotted, and the area under the curve (AUC) was calculated to assess the classification performance of each model. Additionally, the goodness-of-fit of the models was evaluated using the Hosmer-Lemeshow test, and calibration curves were plotted to verify model calibration. Decision curve analysis (DCA) was conducted to assess the clinical utility of the models. Model selection was based on predictive performance and stability criteria, as detailed in the Results section. A performance evaluation chart was created to summarize the overall performance of each model. A comprehensive report was also generated to provide further guidance for clinical application and research purposes.

### 2.4 Statistical analysis

This study analyzed existing data using Python version 3.1.2. Categorical variables were presented as percentages and analyzed using the Chi-square test or Fisher’s exact test. Continuous variables were processed based on their distribution characteristics. Variables following a normal distribution were expressed as mean ± standard deviation (Mean ± SD) and compared using the independent sample t-test. Variables not following a normal distribution were presented as median and interquartile range (Median [IQR]) and analyzed using nonparametric tests. A two-sided P-value of <0.05 was considered statistically significant. As this is an exploratory study, multiple comparison corrections were not performed to avoid excessively reducing the statistical significance level. The aforementioned methods ensure the scientific rigor and reliability of the analysis results.

## 3 Results

### 3.1 Screening of clinical feature variables

**Figure 4:**
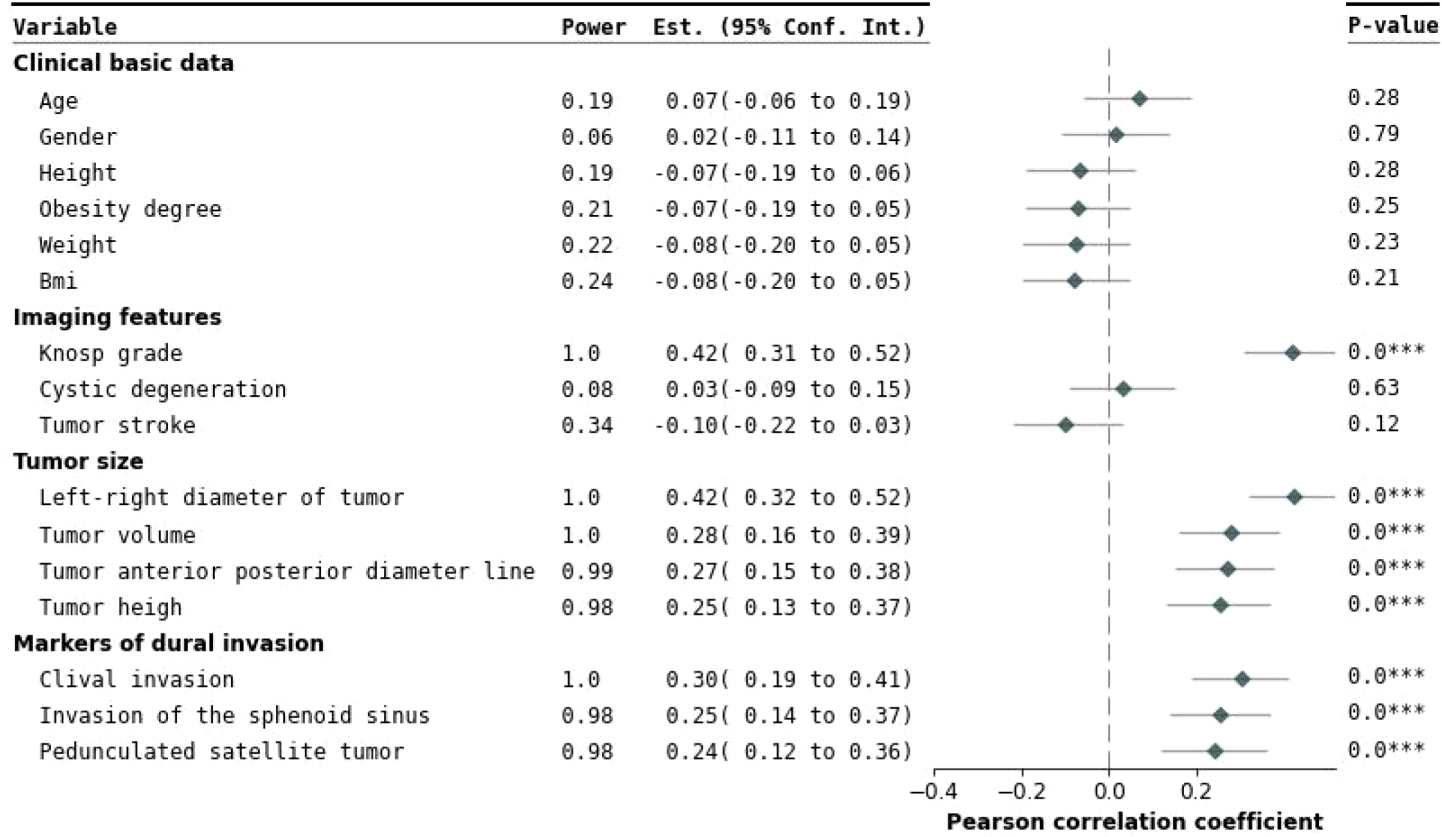
Forest plot of univariate analysis. This figure presents the effects and statistical significance of each variable identified in the univariate analysis. The variables are categorized into clinical baseline information, imaging characteristics, tumor size, and cavernous sinus dural invasion markers. On the left, the regression estimates and their 95% confidence intervals for each variable are displayed, clearly illustrating the independent impact of the variables on cavernous sinus dural invasion. On the right, a Pearson correlation coefficient forest plot evaluates inter-variable correlations. Statistical significance is indicated by P-values, with *P* < 0.05 denoting significant differences. Variables such as Knosp grade, tumor height, tumor anteroposterior diameter, tumor lateral diameter, pedunculated satellite tumor, tumor volume, clival invasion, and sphenoid sinus invasion showed significant associations. ***This indicates that the result is statistically significant (p < 0.05)

A total of 252 pituitary adenoma patients were included in this study, comprising 132 males and 120 females. The univariate analysis (**Table 1**) revealed significant differences in multiple clinical characteristics between the cavernous sinus invasion (CSI) group and the non-invasion group (*P* < 0.05). Specifically, eight variables showed significant differences: tumor anteroposterior diameter, tumor left-right diameter, tumor height, tumor volume, Knosp grade, pedunculated satellite tumor, sphenoid sinus invasion, and clivus invasion (**Figure 4**).

**Table 1:** Baseline characteristics of variables associated with cavernous sinus invasion following transnasal endoscopic pituitary adenoma resection.

Variable		Total	Not	Invasion	P-Value
		252	124	128	
Gender					
	Male	132 (52.4)	66 (53.2)	66 (51.6)	0.89
	Female	120 (47.6)	58 (46.8)	62 (48.4)	
Age, median [Q1,Q3]		50.5 [40.0,59.0]	50.0 [35.8,60.0]	51.0 [43.0,57.2]	0.363
Knosp Grade					
	0-1	64 (25.4)	49 (39.5)	15 (11.7)	<0.001
	2-3a	103 (40.9)	56 (45.2)	47 (36.7)	
	3b-4	85 (33.7)	19 (15.3)	66 (51.6)	
Tumor heigh, median [Q1,Q3]		26.0 [18.6,34.1]	23.6 [15.5,31.9]	28.0 [21.6,36.7]	<0.001
Tumor anterior posterior diameter line, median [Q1,Q3]		19.8 [15.1,24.7]	18.2 [12.6,23.1]	21.0 [17.7,26.0]	<0.001
Left-right diameter of tumor, median [Q1,Q3]		26.3 [21.0,30.7]	23.4 [18.6,27.5]	29.3 [25.4,33.8]	<0.001
Pedunculated satellite tumor					
	No	208 (82.5)	114 (91.9)	94 (73.4)	<0.001
	Yes	44 (17.5)	10 (8.1)	34 (26.6)	
Tumor volume, median [Q1,Q3]		13.5 [6.4,25.9]	9.5 [4.6,18.8]	16.7 [9.6,31.7]	<0.001
Cystic degeneration					
	No	221 (87.7)	110 (88.7)	111 (86.7)	0.772
	Yes	31 (12.3)	14 (11.3)	17 (13.3)	
Tumor stroke					
	No	203 (80.6)	95 (76.6)	108 (84.4)	0.162
	Yes	49 (19.4)	29 (23.4)	20 (15.6)	
Clival invasion					
	No	134 (53.2)	85 (68.5)	49 (38.3)	<0.001
	Yes	118 (46.8)	39 (31.5)	79 (61.7)	
Invasion of the sphenoid sinus					
	No	108 (42.9)	69 (55.6)	39 (30.5)	<0.001
	Yes	144 (57.1)	55 (44.4)	89 (69.5)	
Height, median [Q1,Q3]		163.0 [158.0,170.0]	163.5 [158.0,170.0]	163.0 [158.0,170.0]	0.539
Weight, median [Q1,Q3]		65.0 [57.0,72.0]	65.0 [57.5,75.0]	64.0 [57.0,71.8]	0.318
BMI, median [Q1,Q3]		23.8 [22.0,26.1]	23.9 [22.0,26.5]	23.6 [21.9,25.7]	0.276
Obesity Degree					
	Normal	129 (51.2)	60 (48.4)	69 (53.9)	0.285
	Slim	11 (4.4)	3 (2.4)	8 (6.2)	
	Overweight	76 (30.2)	42 (33.9)	34 (26.6)	
	Obesity	36 (14.3)	19 (15.3)	17 (13.3)	

**Figure 5:**
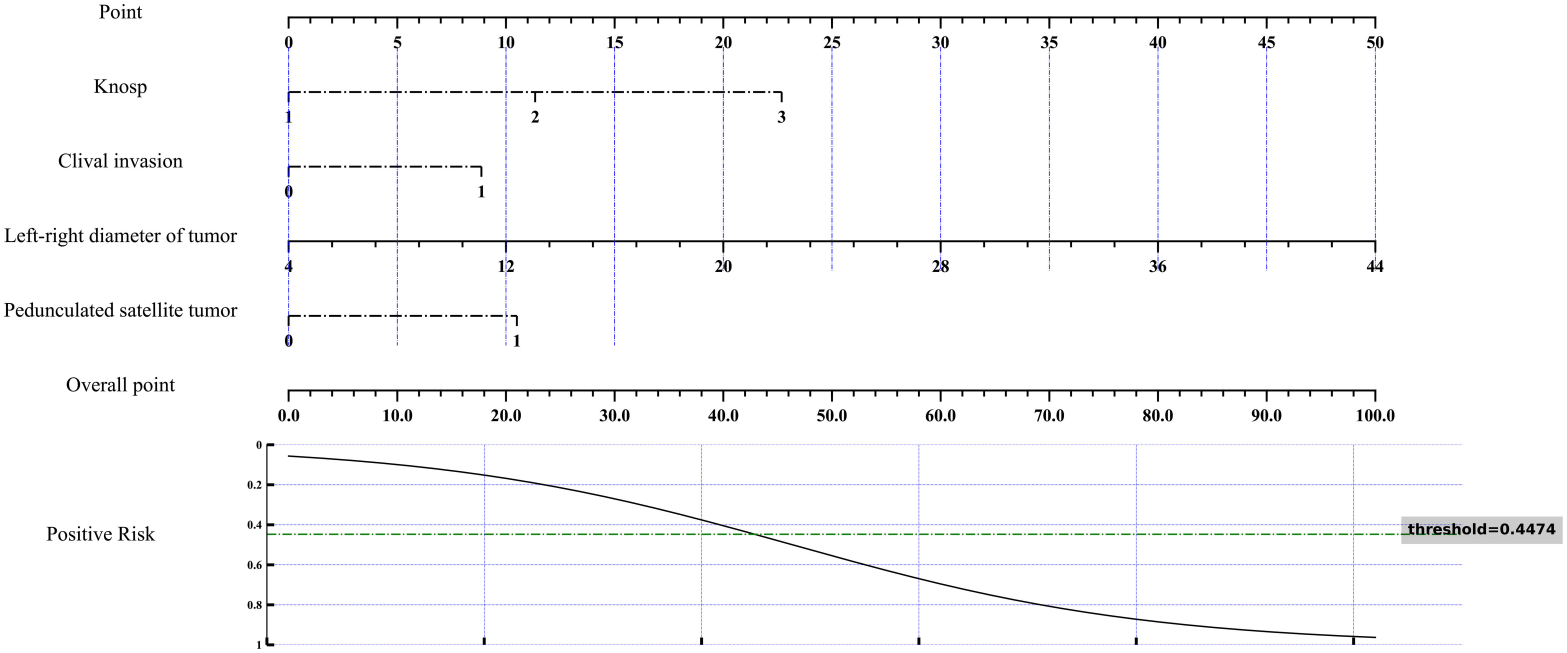
Nomogram for multivariate analysis of cavernous sinus dural invasion. A nomogram was developed based on the results of multivariate analysis to assess the risk of cavernous sinus dural invasion. This nomogram integrates various independent risk factors, providing a comprehensive risk evaluation for cavernous sinus dural invasion. The total score and corresponding risk evaluation curve quantitatively illustrate the influence of each factor on cavernous sinus dural invasion, facilitating individualized prediction and aiding in clinical decision-making.

**Figure 6:**
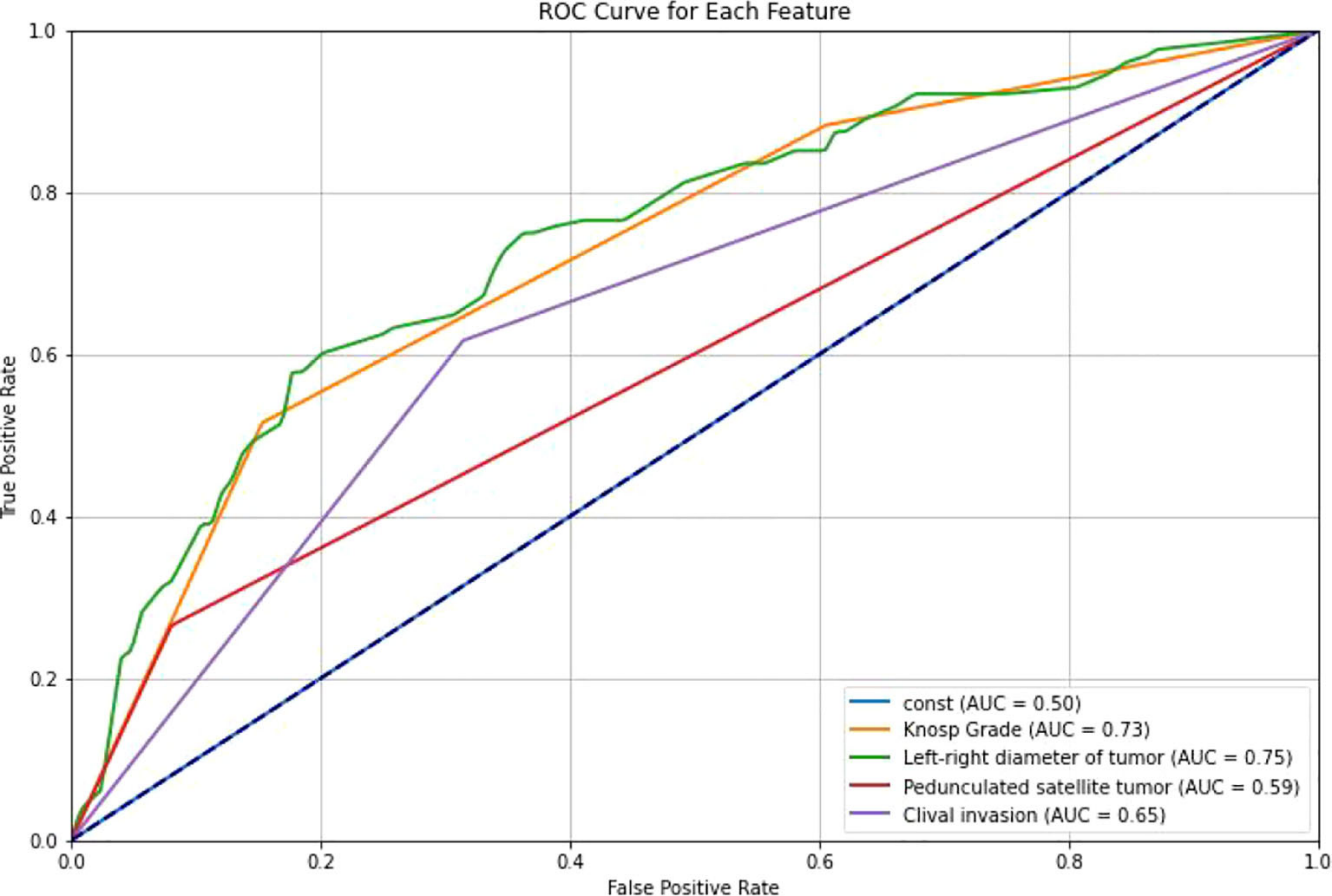
ROC curves of different features. ROC curves were generated for the selected clinical features to evaluate their association with cavernous sinus dural invasion in pituitary adenomas. The results demonstrated that all selected features exhibited varying degrees of predictive performance in univariate analysis, further supporting their significant correlation with cavernous sinus dural invasion.

Further multivariate logistic regression analysis, with a selection threshold of *P* < 0.1, was performed to evaluate the independence and impact of each variable whereas controlling for potential confounding factors (23) (**Figure 5**). The analysis confirmed that Knosp grade, tumor left-right diameter, pedunculated satellite tumor, and clivus invasion were independent influencing factors. These variables were subsequently included in the feature set for constructing machine learning models to predict the risk of cavernous sinus invasion (**Figure 6**).

### 3.2 Prediction models based on clinical features

**Figure 7:**
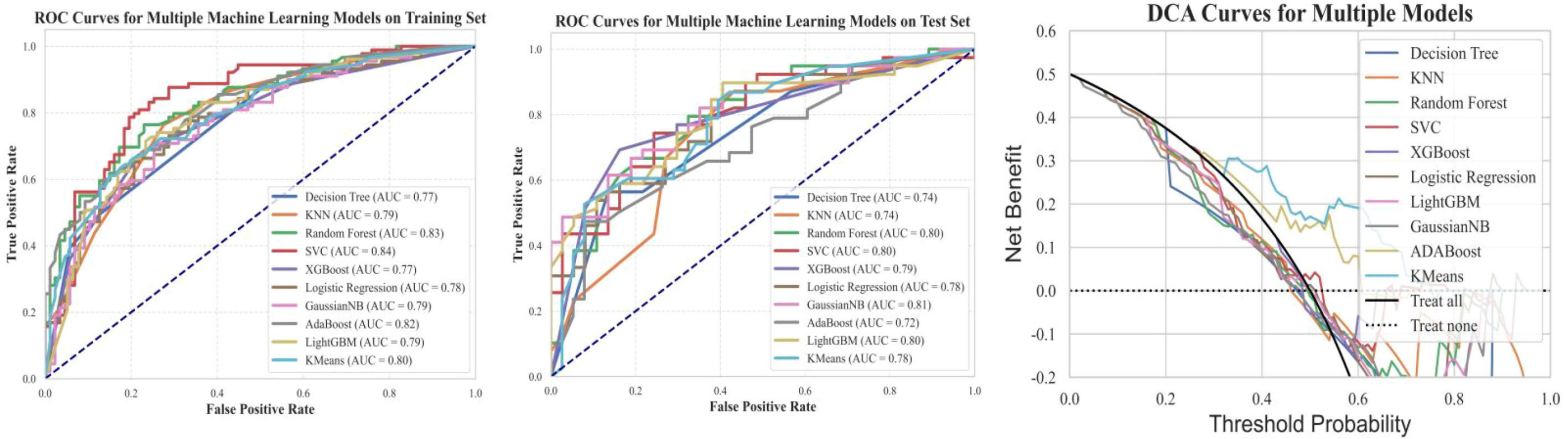
Training set ROC, testing set ROC, and DCA curves for machine learning models based on clinical features. This figure illustrates the Receiver Operating Characteristic (ROC) curves of machine learning models based on clinical features for both the training and testing datasets, along with the corresponding Decision Curve Analysis (DCA) results. The findings indicate that the models exhibit good classification performance on both datasets. Additionally, the DCA curves suggest that the models have potential clinical utility.

Prediction models were constructed using clinical features and ten different machine learning algorithms. ROC curves were plotted for both the training and testing datasets, and the AUC was calculated to evaluate model performance (**Figure 7**). Calibration curves were also analyzed to assess the goodness-of-fit and generalization ability of the models, verifying their stability and consistency across datasets. The results (**Table 2**) show that prediction models based on clinical features effectively predict cavernous sinus dural invasion in pituitary adenomas, providing a foundational reference for subsequent integrated radiomic feature modeling.

**Table 2:** Performance comparison and consistency testing of ten machine learning models based on clinical features.

Model	Train AUC	Test AUC	ACC	TPR(SEN)	FPR	PPV	SPE	NPV	FSC	Brier Score	Hosmer-Lemeshow statistic	Hosmer-Lemeshow p-value
DT	0.77	0.74	65.79	0.87	0.57	0.62	0.43	0.76	0.72	0.20	1.93	0.98
KNN	0.79	0.74	72.37	0.87	0.43	0.68	0.57	0.81	0.76	0.21	12.65	0.12
RF	0.83	0.80	71.05	0.79	0.38	0.69	0.62	0.74	0.74	0.19	6.69	0.57
SVC	0.84	0.80	69.74	0.82	0.43	0.67	0.57	0.75	0.74	0.20	5.98	0.65
XGBoost	0.77	0.79	0.72	0.77	0.32	0.71	0.68	0.74	0.74	0.22	12.71	0.12
LR	0.78	0.78	0.74	0.85	0.38	0.70	0.62	0.79	0.77	0.20	11.90	0.16
GNB	0.77	0.81	0.71	0.82	0.41	0.68	0.59	0.76	0.74	0.18	8.35	0.40
LightGBM	0.79	0.80	0.70	0.77	0.38	0.68	0.62	0.72	0.72	0.19	9.06	0.34
AdaBoost	0.82	0.72	0.64	0.66	0.37	0.64	0.63	0.65	0.65	0.23	4.63	0.80
Kmean	0.80	0.78	0.67	0.61	0.26	0.70	0.74	0.65	0.65	0.20	8.24	0.41

Among the evaluated models, GNB demonstrated the most favorable calibration and discrimination characteristics. It achieved the highest test AUC (0.81), indicating excellent discriminatory power, and recorded the lowest Brier score (0.18), reflecting superior accuracy in probability estimation. Furthermore, the Hosmer-Lemeshow test yielded a non-significant p-value (p=0.40), suggesting good agreement between predicted and observed outcomes. The model also showed balanced classification performance, with a sensitivity of 0.82 and an F1-score of 0.74. Given its strong discriminative ability, optimal calibration, and simplicity in implementation, GNB was selected as the preferred predictive model in this study.

### 3.3 Machine learning models based on radiomic features

**Figure 8:**
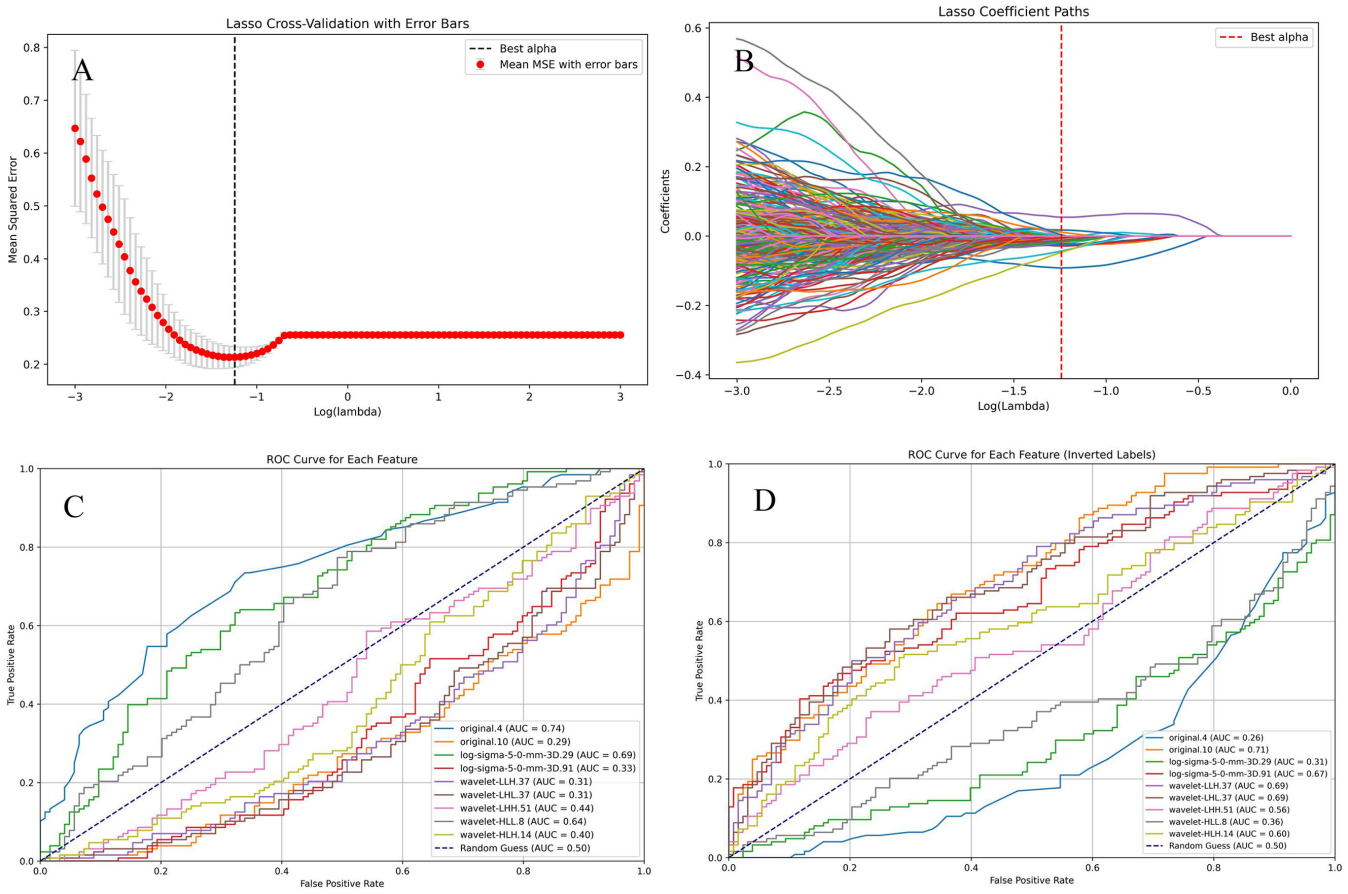
Selection of radiomic features. **(A, B)**. Lasso regression was used to select radiomic features with statistical significance. **(C, D)**. The ROC curve for each selected feature.

Through manual extraction of pituitary adenoma imaging features from T2 coronal images using 3D Slicer, a total of 1073 imaging features were obtained, covering morphological, texture, and advanced radiomic characteristics. Lasso regression was applied to select 9 statistically significant features, which were further filtered using univariate regression analysis. 7 imaging features, including original.4, original.10, log-sigma-5-0-mm-3D.29, log-sigma-5-0-mm-3D.91, wavelet-LLH.37, wavelet-LHL.37, and wavelet-HLL.8, were included for study and model construction (**Figure 8**).The seven radiomic features used in the final model describe different characteristics of the tumor, such as the overall brightness and contrast (first-order features), the texture and patterns inside the tumor (texture features), and information from filtered images that highlight edges or fine details (LoG and wavelet features). These features help to capture both the appearance and internal structure of the tumor on imaging.

**Figure 9:**
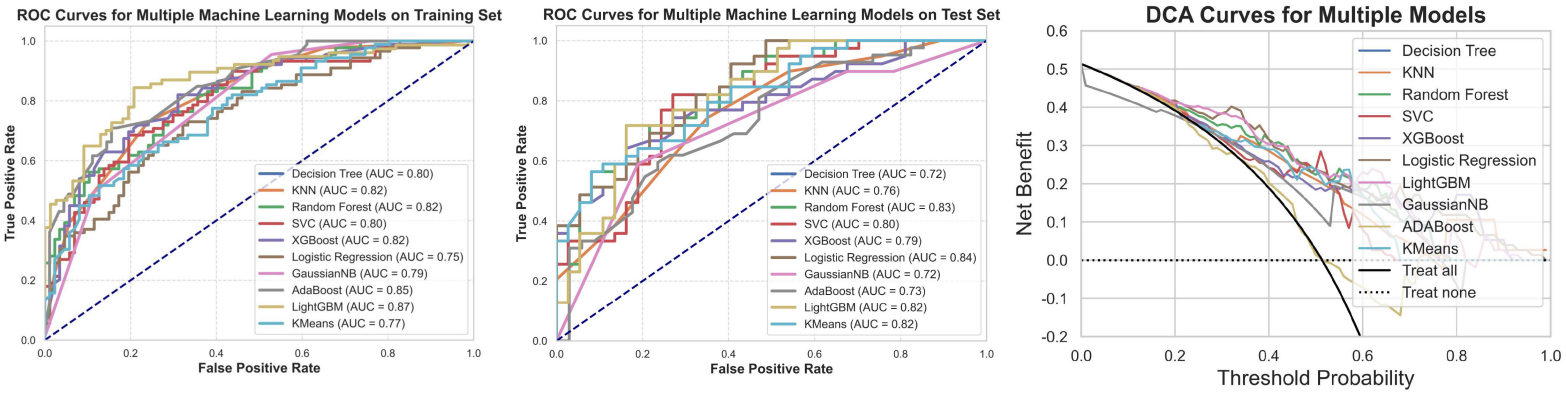
ROC, test set ROC, and DCA curves for the machine learning model based on radiomic features. This figure illustrates the ROC curves for the machine learning model constructed based on radiomic features in both the training and test sets, along with the corresponding Decision Curve Analysis (DCA) results. The results show that the model based on radiomic features demonstrates good classification ability in both the training and test sets, and the DCA curve indicates its potential clinical applicability.

Based on the selected radiomic features, predictive models were established using ten different machine learning algorithms. ROC curves were plotted for both the training and testing sets, and AUC was calculated to evaluate model performance. Additionally, calibration curves were analyzed to assess model fitting and generalization ability (**Figure 9**).

The results indicate that the models based on radiomic features exhibited good classification ability in both the training and testing sets. The performance of these models was generally superior to models based solely on clinical features, particularly in key metrics such as AUC, sensitivity, and specificity. This highlights the significant value of radiomic features in predicting cavernous sinus dural invasion in pituitary adenomas and provides strong support for subsequent integrated clinical-radiomic modeling (**Table 3**).

**Table 3:** Performance comparison and consistency test results of ten machine learning models built on radiomic features.

Model	Train AUC	Test AUC	ACC	TPR(SEN)	FPR	PPV	SPE	NPV	FSC	Brier score	Hosmer-Lemeshow statistic	Hosmer-Lemeshow p-value
DT	0.80	0.72	61.84	0.90	0.68	0.58	0.32	0.75	0.71	0.23	inf	0.00
KNN	0.82	0.76	69.74	0.74	0.35	0.69	0.65	0.71	0.72	0.20	5.05	0.75
RF	0.82	0.83	71.05	0.74	0.32	0.71	0.68	0.71	0.73	0.18	7.48	0.49
SVC	0.80	0.80	77.63	0.82	0.27	0.76	0.73	0.79	0.79	0.20	10.17	0.25
XGBoost	0.74	0.85	0.74	0.69	0.22	0.77	0.78	0.71	0.73	0.17	9.67	0.29
LR	0.82	0.79	0.67	0.79	0.46	0.65	0.54	0.71	0.71	0.19	9.89	0.27
GNB	0.74	0.81	0.76	0.82	0.46	0.65	0.54	0.74	0.73	0.24	103.54	0.00
LightGBM	0.87	0.82	0.72	0.79	0.35	0.70	0.65	0.75	0.75	0.17	6.20	0.63
AdaBoost	0.85	0.73	0.67	0.60	0.24	0.76	0.76	0.60	0.67	0.21	3.24	0.92
Kmean	0.77	0.82	0.72	0.79	0.35	0.70	0.65	0.75	0.75	0.20	11.83	0.16

Among the ten models evaluated, LightGBM demonstrated the best overall calibration, as reflected by the lowest Brier score (0.17) and a non-significant Hosmer-Lemeshow test (p = 0.63), indicating good agreement between predicted and observed outcomes. The model also showed strong discriminative ability with a test AUC of 0.82, along with balanced sensitivity (0.77) and F1-score (0.72). Although its false positive rate was slightly higher compared to some other models, LightGBM’s combination of calibration and discrimination makes it the preferred model for this study.

### 3.4 Machine learning models constructed based on clinical features combined with radiomic features

**Figure 10:**
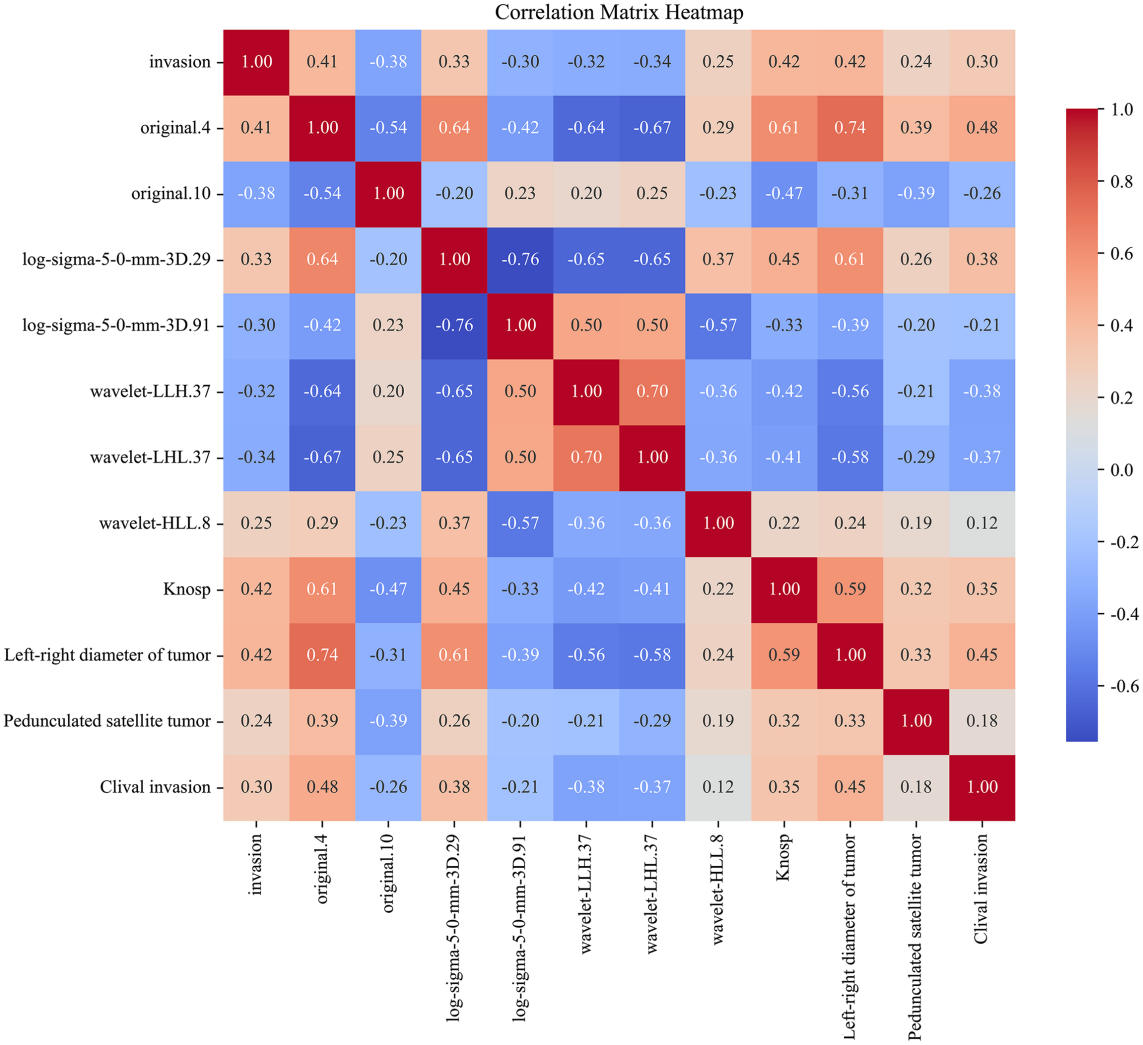
Correlation heatmap of clinical features and radiomic features. This heatmap illustrates the correlation between the selected clinical and radiomic features and cavernous sinus invasion. The correlation coefficients are calculated, with the color intensity in the heatmap reflecting the strength of the correlation. Red indicates a positive correlation, whereas blue indicates a negative correlation.

**Figure 11:**
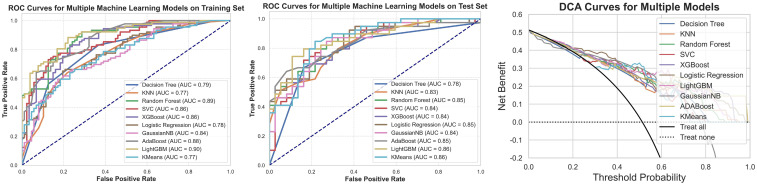
ROC curves, test set ROC curves, and DCA curves for the machine learning models constructed using clinical and radiomic features. This figure presents the ROC curves for the machine learning model based on clinical and radiomic features for both the training and testing datasets, along with the corresponding DCA results. The findings indicate that the model built on clinical and radiomic features exhibits strong classification ability in both datasets, and the DCA curve demonstrates its potential clinical utility.

The correlation between the clinical and radiomic features selected earlier was assessed, and a correlation heatmap (**Figure 10**) was plotted to illustrate the interrelationships among the variables. Ten different machine learning algorithms were then used to construct prediction models, and their performance in predicting cavernous sinus invasion in pituitary adenomas was evaluated (**Figure 11**). ROC curves were generated to assess the classification performance of the models, and DCA curves were employed to evaluate their potential clinical applicability.

Among the ten machine learning algorithms evaluated (**Table 4**), the model constructed using the LightGBM algorithm exhibited the best overall performance. It achieved the highest AUC values in both the training (AUC = 0.90) and testing (AUC = 0.86) sets, with a prediction accuracy of 0.76, and performed favorably across other performance metrics. When comparing the optimal models established using three different feature sets, the model integrating both clinical and radiomic features demonstrated the best predictive performance and showed superior capability in the preoperative prediction of medial wall invasion of the cavernous sinus.

**Table 4:** Performance and consistency test results of machine learning models built using clinical and radiomic features.

Model	Train AUC	Test AUC	ACC	TPR	FPR	PPV	SPE	SEN	NPV	FSC	Brier score	Hosmer-Lemeshow statistic	Hosmer-Lemeshow p-value
DT	0.79	78.00	71.05	0.87	0.46	0.67	0.54	0.87	0.80	0.76	0.19	inf	0.00
KNN	0.77	0.83	71.05	0.67	0.24	0.74	0.76	0.67	0.68	0.70	0.18	4.67	0.79
RF	0.89	0.85	76.32	0.77	0.24	0.77	0.76	0.77	0.76	0.77	0.17	6.22	0.62
SVC	0.86	0.84	78.95	0.82	0.24	0.78	0.76	0.82	0.80	0.80	0.20	13.55	0.09
XGBoost	0.86	0.84	0.74	0.87	0.41	0.69	0.59	0.87	0.81	0.77	0.17	5.74	0.68
LR	0.78	0.85	0.74	0.74	0.27	0.74	0.73	0.74	0.73	0.74	0.16	8.65	0.37
GNB	0.76	0.84	0.79	0.87	0.30	0.76	0.70	0.87	0.84	0.81	0.19	140.56	0.00
LightGBM	0.90	0.86	0.76	0.85	0.32	0.73	0.68	0.85	0.81	0.79	0.16	10.02	0.26
AdaBoost	0.88	0.85	0.74	0.74	0.27	0.74	0.73	0.74	0.73	0.74	0.17	6.31	0.61
Kmean	0.77	0.86	0.82	0.85	0.22	0.80	0.78	0.85	0.83	0.83	0.19	16.26	0.04

**Figure 12:**
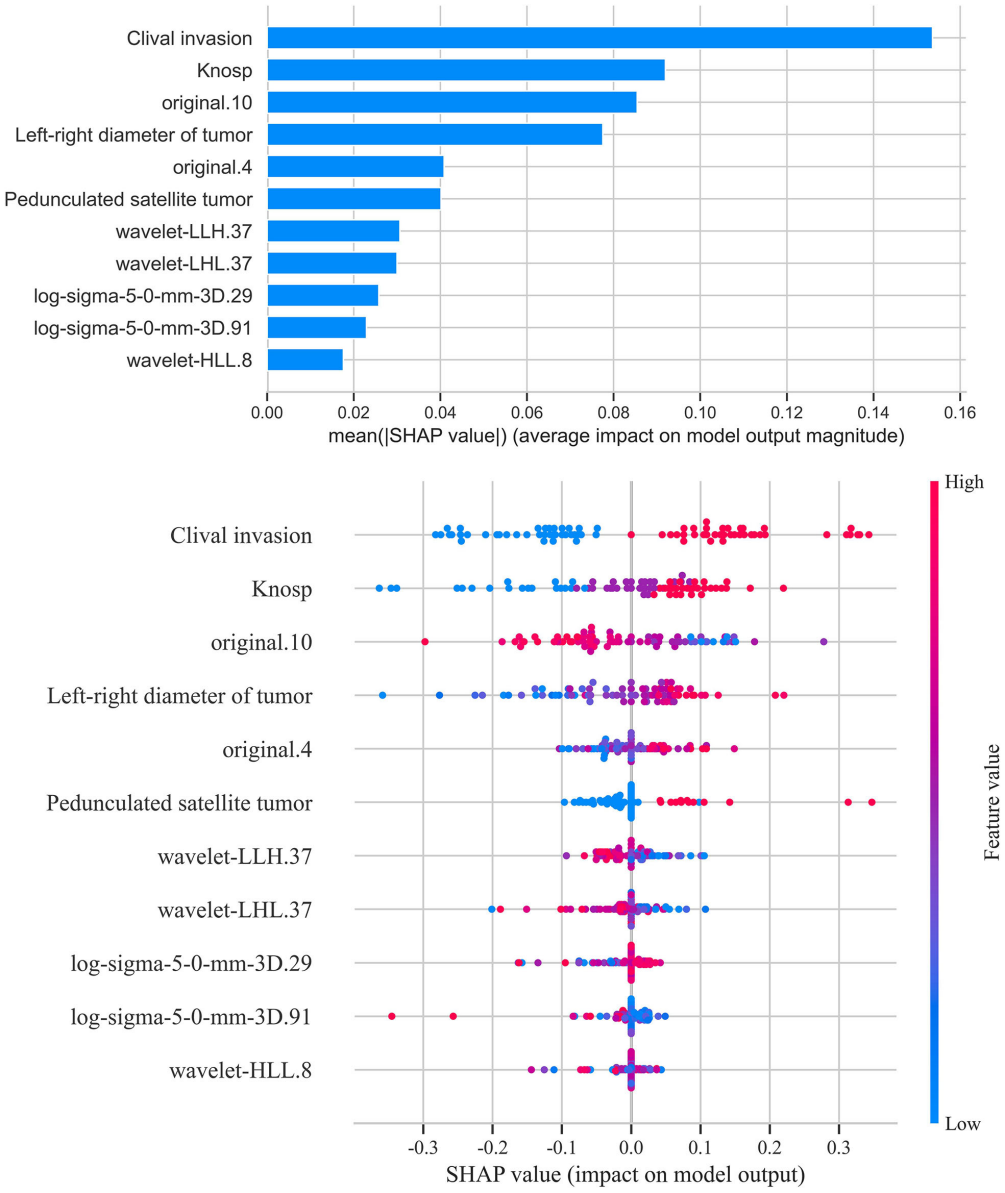
SHAP score analysis - bar and scatter plots. This figure presents the SHAP (SHapley Additive exPlanations) analysis results for the LightGBM model. The bar plot shows the contribution of each feature to the model’s prediction outcomes, ranking the features by importance. The scatter plot reveals the association between feature values and SHAP values, indicating how variations in feature values influence the prediction results. Features such as sphenoid sinus invasion, tumor transverse diameter, Knosp grade, and presence of pedunculated satellite tumors emerged as significant predictors, with their values showing positive correlations with the likelihood of cavernous sinus dural invasion.

SHapley Additive exPlanations (SHAP) analysis (**Figure 12**) was conducted to evaluate feature importance and trend changes in the LightGBM model. The results showed that cavernous sinus invasion, tumor lateral diameter, Knosp grade, pedunculated satellite tumor, and the radiomic feature original.10 were significantly correlated with cavernous sinus invasion in the LightGBM model. Furthermore, cavernous sinus invasion, presence of a satellite tumor, higher Knosp grade, and larger tumor lateral diameter were positively correlated with the diagnostic outcome of cavernous sinus invasion.

In addition to clinical variables, several radiomic features showed significant contributions to the prediction of cavernous sinus invasion in the LightGBM model. Features such as original.4 and original.10 reflect intensity distribution and texture regularity, respectively, and were positively associated with the predicted probability of invasion. Texture-related features extracted from filtered images, including log-sigma-5-0-mm-3D.29 and log-sigma-5-0-mm-3D.91, describe heterogeneity and complexity within the tumor and also showed a positive association. Moreover, wavelet-transformed features such as wavelet-LHL.37, wavelet-LLH.37, and wavelet-HLL.8 contributed additional information related to fine textural patterns and asymmetry, and were similarly associated with higher invasion probability. These results suggest that tumors with greater internal heterogeneity and complex texture patterns tend to be more invasive on imaging.

## 4 Discussion

This study demonstrated that integrating radiomic and clinical features significantly improves the preoperative prediction of cavernous sinus dural invasion in pituitary adenomas. The combined LightGBM model achieved the best predictive performance, confirming the complementary value of quantitative imaging features and clinical parameters. These findings support the growing application of radiomics in neurosurgery, providing a non-invasive approach for assessing tumor invasiveness and aiding surgical planning.CSI in pituitary adenomas is characterized by the destruction and infiltration of the medial wall of the cavernous sinus, with intraoperative observation of medial wall damage serving as a key diagnostic criterion. Although some recent studies have questioned the clinical significance of cavernous sinus dural invasion (5), the prevailing view still recognizes cavernous sinus dural invasion as a critical marker of pituitary adenoma aggressiveness (24). For patients with cavernous sinus dural invasion, the presence of tumor tissue embedded within the dura significantly increases the risk of recurrence. Therefore, accurately assessing cavernous sinus dural invasion preoperatively and tailoring surgical strategies accordingly is crucial for reducing recurrence rates in pituitary adenomas. Despite the high resolution of 3D SPACE imaging, it remains challenging to definitively evaluate medial wall disruption or its adherence to the internal carotid artery wall (9). Consequently, Knosp grading is often used clinically to assess cavernous sinus invasion in pituitary adenomas (25, 26). However, a meta-analysis revealed that Knosp grading is not entirely reliable for this purpose (27). Niu and colleagues reported that 85.4% of Knosp grade 2 pituitary adenomas and 34.3% of Knosp grade 3 adenomas lacked evidence of CSI during surgery (28), suggesting that Knosp grading may not fully reflect the actual incidence of cavernous sinus dural invasion. To address this limitation, Hong et al. (29) proposed the concept of invasion pathways based on the distinct structures of the dura surrounding the pituitary gland. They proposed novel imaging markers, such as pedunculated satellite tumors and interdural tumors, as indicators of cavernous sinus dural invasion, offering new perspectives and evaluation criteria for the study of cavernous sinus dural invasion in pituitary adenomas.

The results of this study show that pedunculated satellite tumors, clival invasion, Knosp grading, and tumor transverse diameter are significant predictors of meningeal invasion in pituitary adenomas. Pedunculated satellite tumors, as an important imaging marker for cavernous sinus meningeal invasion, hold considerable predictive weight in machine learning models, making them a key reference for preoperative evaluation. However, only some pituitary adenomas form pedunculated satellite tumors during invasion, whereas most tumors directly breach the cavernous sinus inner wall, which explains the moderate statistical performance of pedunculated satellite tumors. The invasion of the clivus is closely associated with medial wall invasion of the cavernous sinus (30). This phenomenon may be explained from a biomechanical perspective. The growth direction of pituitary adenomas is influenced by multiple factors, including the stability of the surrounding dura mater and bony structures. Generally, pituitary adenomas tend to invade the diaphragma sellae and cavernous sinus preferentially (3). Once the medial wall of the cavernous sinus and adjacent bony structures are compromised, the clivus—owing to its relatively thin anatomical composition—often becomes the next target of tumor invasion. Therefore, the presence of clival invasion on preoperative imaging may indicate that the tumor has already breached the medial wall of the cavernous sinus (26, 31).Tumor transverse diameter reflects cavernous sinus invasion and is associated with inner wall damage (32, 33). Radiomics, which extracts high-dimensional features from imaging data, provides additional information on tumor invasion and biological behavior (16). This study found that on CE-T2 MR images, features such as wavelet-LLH.37 and wavelet-LHL.37 exhibited significantly reduced mean MMC values in tumors with meningeal invasion, indicating higher tumor heterogeneity and invasiveness. The sphericity value also proved important in predicting invasiveness, with larger values correlating with less invasiveness. Quantifying imaging features enhances preoperative evaluation by revealing tumor heterogeneity and biological characteristics. The radiomic texture features identified in this study, particularly wavelet-LLH.37 and wavelet-LHL.37, may reflect differences in tumor consistency. Prior research has linked MRI-based texture parameters with intraoperative tumor hardness and surgical difficulty. These findings suggest that texture-based radiomic features could provide a non-invasive surrogate for assessing tumor consistency, with potential implications for preoperative planning and intraoperative strategy (14, 34).

This study has several limitations. First, its retrospective single-center design may introduce selection bias and limit generalizability. The relatively small sample size could also increase the risk of overfitting, despite cross-validation. Moreover, the use of a single imaging sequence (T2-weighted MRI) may restrict the comprehensiveness of radiomic feature extraction. Future studies should validate the proposed model in larger, multicenter cohorts and explore the integration of multi-sequence MRI or multi-omics data to enhance robustness and clinical applicability.

## 5 Conclusion

By integrating clinical data with radiomics features, the machine learning model based on the LightGBM algorithm demonstrated exceptional performance in preoperatively predicting the invasiveness of pituitary adenomas into the dura mater. The model achieved an AUC of 0.86 and an ACC of 0.76, highlighting its significant predictive advantages. Additionally, the presence of pedunculated satellite tumors, as a novel radiological marker for pituitary adenomas, played a critical role in predicting invasiveness, offering new insights for the preoperative identification of invasive pituitary adenomas.

## Data Availability

The raw data supporting the conclusions of this article will be made available by the authors, without undue reservation.
